# A metabolome‐based core hybridisation strategy for the prediction of rice grain weight across environments

**DOI:** 10.1111/pbi.13024

**Published:** 2018-11-12

**Authors:** Zhiwu Dan, Yunping Chen, Yanghong Xu, Junran Huang, Jishuai Huang, Jun Hu, Guoxin Yao, Yingguo Zhu, Wenchao Huang

**Affiliations:** ^1^ State Key Laboratory of Hybrid Rice Key Laboratory for Research and Utilization of Heterosis in Indica Rice The Yangtze River Valley Hybrid Rice Collaboration & Innovation Center College of Life Sciences Wuhan University Wuhan China; ^2^ School of Life and Science Technology Hubei Engineering University Xiaogan China

**Keywords:** prediction, metabolic markers, partial least squares regression, core hybrids, grain weight, rice (*Oryza sativa*)

## Abstract

Marker‐based prediction holds great promise for improving current plant and animal breeding efficiencies. However, the predictabilities of complex traits are always severely affected by negative factors, including distant relatedness, environmental discrepancies, unknown population structures, and indeterminate numbers of predictive variables. In this study, we utilised two independent F_1_ hybrid populations in the years 2012 and 2015 to predict rice thousand grain weight (TGW) using parental untargeted metabolite profiles with a partial least squares regression method. A stable predictive model for TGW was built based on hybrids from the population in 2012 (*r* = 0.75) but failed to properly predict TGW for hybrids from the population in 2015 (*r* = 0.27). After integrating hybrids from both populations into the training set, the TGW of hybrids could be predicted but was largely dependent on population structures. Then, core hybrids from each population were determined by principal component analysis and the TGW of hybrids in both environments were successfully predicted (*r* > 0.60). Moreover, adjusting the population structures and numbers of predictive analytes increased TGW predictability for hybrids in 2015 (*r* = 0.72). Our study demonstrates that the TGW of F_1_ hybrids across environments can be accurately predicted based on parental untargeted metabolite profiles with a core hybridisation strategy in rice. Metabolic biomarkers identified from early developmental stage tissues, which are grown under experimental conditions, may represent a workable approach towards the robust prediction of major agronomic traits for climate‐adaptive varieties.

## Introduction

The utilisation of hybrid vigour in crops and livestock has achieved substantial production improvement over the past decades. However, simultaneously, the breeding of an elite hybrid requires many hybridisations and phenotypic identification tasks year after year. Randomness in breeding programmes severely affects breeding efficiency, and much room for improvement exists in the current pace of hybrid breeding.

Marker‐assisted selection and genomic selection are expected to improve breeding efficiency, and different types of biomarkers are used to predict hybrid performances. However, several obstacles must be overcome before the markers can be appropriately applied to accelerate breeding programmes. The first obstacle is the relatedness between training and validation sets in the cross‐validation procedure (Schulthess *et al*., 2017; Wray *et al*., 2013). In predicting grain yield and grain dry matter content in hybrid maize with DNA markers and transcriptome‐based distance, predictabilities of corresponding hybrids were high (0.74–0.99) with parents included in the training set; whereas predictabilities dropped dramatically (0.29–0.56) when no parent was part of the training set (Zenke‐Philippi *et al*., 2016). Similar findings are also reported in the prediction of yield and disease resistance in hybrid wheat (Gowda *et al*., 2014b; Zhao *et al*., 2015). Thus, distant relatedness between test and estimation sets can severely decrease prediction accuracy.

The second barrier is environmental discrepancies between training and validation sets (Schulthess *et al*., 2017). In hybrid rye, not only the grade of relatedness affected predictabilities but also the number of evaluation locations and testing years of phenotypic data included in the training set influenced prediction accuracy (Wang *et al*., 2014). In addition, in hybrid maize, predictabilities were low when phenotypes were predicted separately; however, predictabilities benefited when the environmental structure was considered in the training set (Windhausen *et al*., 2012). Another study in wheat reported that correction for spatial variations (across locations or year to year) was crucial to increase predictabilities, and the best prediction for hybrids across three environments appeared when phenotypic data from different years were in the training set (Lado *et al*., 2013). Hence, environmental effects should be considered in employing marker‐based prediction. In addition, other factors including population structures and numbers of predictive variables also have large effects on predictabilities (Guo *et al*., 2016; Slavov *et al*., 2014; Technow *et al*., 2014; Windhausen *et al*., 2012; Xu *et al*., 2017).

Metabolomics‐assisted breeding has been suggested to accelerate breeding processes, particularly for developing disease resistance and herbicide/salinity‐tolerant crops (Fernie and Schauer, 2009). The application potential of metabolic markers became apparent when 73% of identified metabolites demonstrated significant correlations with at least one whole‐plant phenotypic trait in tomato (Schauer *et al*., 2006). Then, metabolites (tyrosine, threonine, and fructose, among others) were found to be robust predictive markers for susceptibility to black spot bruising and chip quality across environments in potato (Steinfath *et al*., 2010). Notably, in the prediction of seven complex traits in hybrid maize with both whole‐genome and metabolic prediction models, predictabilities ranged from 0.72 to 0.81 for SNPs and from 0.60 to 0.80 for metabolites (Riedelsheimer *et al*., 2012). The predictabilities for leaf metabolites as predictors that were only slightly lower than those for SNPs were unexpected since only 130 metabolites were used in the model, whereas the number of SNPs was 56 110, which implies powerful predictive ability of metabolites for predicting highly polygenic traits.

In this study, rice grain weight of two independent hybrid populations was measured and parental untargeted metabolite profiles were obtained to respond to challenges in current marker‐assisted hybrid crop breeding programmes, including population relatedness, environmental effects, population structures and numbers of predictive variables.

## Results

### Hybrid populations for the prediction of rice grain weight

**Figure 1:**
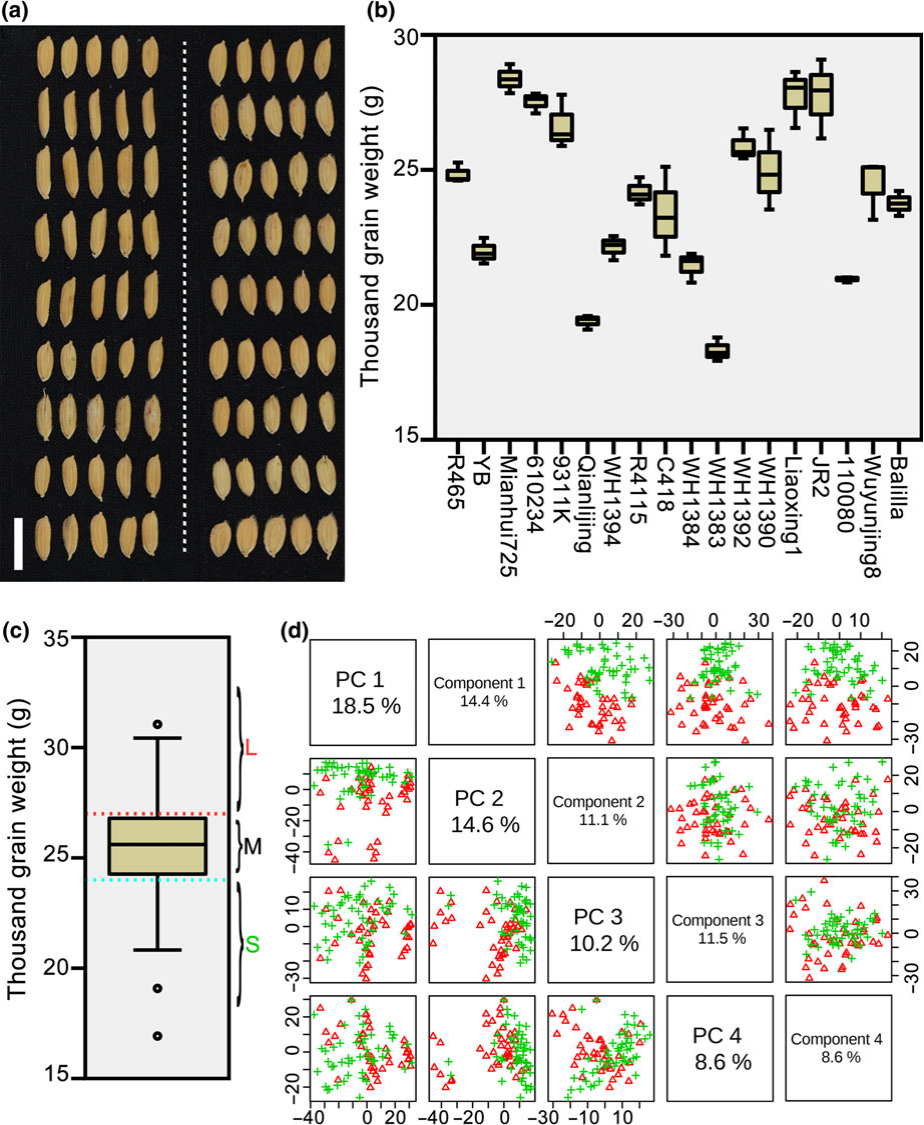
Grain performance and selection of metabolic markers for thousand grain weight. Eighteen representative rice cultivars (including typical *indica*, intermediate types and typical *japonica*) were selected as parents of a hybrid population with a complete diallel cross design in 2012. These cultivars had different grain performance (a) and thousand grain weight (b). The order of grains displayed in (a) is the same as that of grain weight in (b). The white bar in (a) represents 1 cm. (c) Box plot for thousand grain weight (TGW) of hybrids from the population in 2012. The hybrid population was divided into three subgroups according to values of TGW, namely, large (>27 g/1000 grains), medium (24–27 g/1000 grains), and small (<24 g/1000 grains). (d) Distribution of hybrids with large and small TGW. PCA and PLS‐DA were performed with predictive analytes for hybrids with large and small TGW. Pairwise score plots between the top four components of PCA and PLS‐DA are shown on the left and right respectively. Green crisscrosses represent hybrids with small TGW, and red triangles represent hybrids with large TGW.

To identify a workable prediction strategy for grain weight in hybrid rice, this study used two hybrid populations, one in 2012 and one in 2015. For the first population, 18 representative *indica* and *japonica* (*Oryza sativa* L. ssp.) were the parents, and a complete diallel cross‐design was adopted in the hybridisation procedure. The selected 18 inbred lines, which consisted of typical *indica*, intermediate types and typical *japonica*, had distinct grain performance (Figure 1a) and different thousand grain weight (TGW) values (Figure 1b). For the second population, 106 recombinant inbred lines (F_5_) from crosses between the above parents and other cultivars were the male parents, and a Honglian‐type cytoplasmic male sterile line Yuetai A was the female parent. To enlarge environmental effects on phenotypes, phenotypic data of TGW for the first population were measured in 2012, and those of the second population were recorded in 2015 in a different field with narrower planting spaces (Table S1).

### Selection of metabolic markers for grain weight

Our previous report demonstrated that the parental metabolome manifested promising applications in the prediction of multiple agronomic traits in hybrid rice (Dan *et al*., 2016). Thus, untargeted liquid chromatography‐mass spectrometry (LC‐MS) analysis was applied to parental seedlings of the two populations to obtain their metabolite profiles (Table S2), and the mean relative abundances of all identified analytes between every two parents were calculated as predictive analytes for corresponding hybrids. Then, according to values of TGW, hybrids of the first population were divided into three subgroups, namely, large (>27 g/1000 grains), medium (24–27 g/1000 grains), and small (<24 g/1000 grains; Figure 1c). The result of principal component analysis (PCA, a type of unsupervised clustering method; Jolliffe, 2014) based on predictive analytes showed separate clustering trends between hybrids with large and small TGW (Figure 1d), particularly on Component 2, which suggested metabolic similarity of same group hybrids. To identify analytes that might be potential markers for prediction of TGW, we performed partial least squares‐discriminate analysis (PLS‐DA): a supervised method that is always used for classification in metabolomics (Barker and Rayens, 2003; Gardinassi *et al*., 2017). Hybrids from large and small groups distributed almost separately on the first component (Figure 1d). In addition, based on values of variable importance in the projection (VIP) of Component 1 (Table S3), we chose the VIP cutoff of 1.5 (usually at 1.0) as a relatively stringent level to screen predictive variables (with 458 analytes).

### Population relatedness, environmental effects, and population structures severely affect predictability

**Figure 2:**
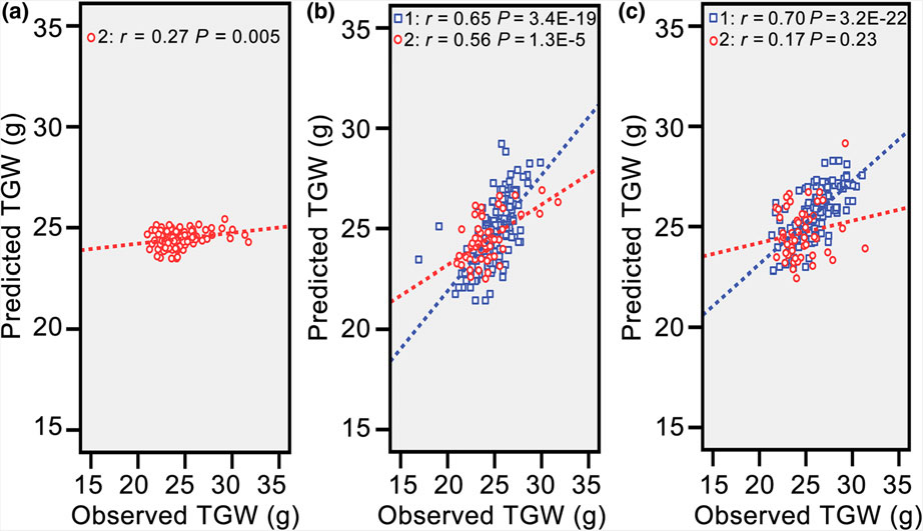
Population relatedness, environmental effects, and population structures had negative effects on the predictability of thousand grain weight. (a) Correlation between the observed and predicted thousand grain weight with all hybrids from the population in 2012 as the training set. (b) Correlation between the observed and predicted thousand grain weight with the first half of the population in 2012 and half of the hybrids from the population in 2015 as the training set. (c) Correlation between the observed and predicted thousand grain weight when reversing the validation set in (b) as the training set. Red circles represent hybrids from the population in 2015, and blue squares represent hybrids from the population in 2012.

Before conducting predictions, we performed PCA for predictive analytes of the two populations to check population relatedness. The distribution plot of hybrids from the two populations showed almost no overlap (Figure S1); thus, the two populations used in this study were metabolically distinct and appropriate for the cross‐validation process (Wray *et al*., 2013). Partial least squares (PLS) was applied to identify connections between metabolic markers and TGW of hybrids in 2012. No more than 17 latent factors could be extracted, and the highest adjusted *R*‐square value (0.569) emerged with five latent factors. After fixing the number of the latent factor at the highest adjusted *R*‐square value, PLS was conducted again, and parameters of all independent variables were used to build an equation for TGW of hybrids in 2015. Although a significant correlation was found between predicted and observed TGW, the predictability was low (*r* = 0.27, *P *=* *0.005; Figure 2a). Therefore, prediction of TGW for new hybrids was not workable with this method because of distant relatedness and environmental discrepancies.

One favourable strategy to realise reliable prediction for new hybrids may be integrating these inevitable negative factors into the training model (Lado *et al*., 2013; Wang *et al*., 2014). Hence, the first part of the population in 2012 and randomly selected hybrids from the population in 2015 were grouped as the training set, and the remaining hybrids in 2012 and 2015 were treated as the validation set. Unexpectedly, high predictability of TGW for hybrids in 2012 (*r* = 0.65, *P *=* *3.4E‐19) and in 2015 (*r* = 0.56, *P *=* *1.3E‐5) was achieved simultaneously (Figure 2b). However, when reversing the validation set as the training set (second part of the population in 2012 and the other unselected hybrids in 2015), the correlation was no longer significant between the predicted and observed values for hybrids in 2015 (*r* = 0.17, *P *=* *0.23; Figure 2c). However, the predictability for hybrids in 2012 was as high as 0.70 (*P *=* *3.2E‐22). Therefore, these attempts demonstrated that including a certain number of new hybrids in the training set can improve predictability; and population structures of training and validation sets also have large influences on predictive outcomes.

### Core hybrids from both populations in the training set can realise high predictabilities across environments

**Figure 3:**
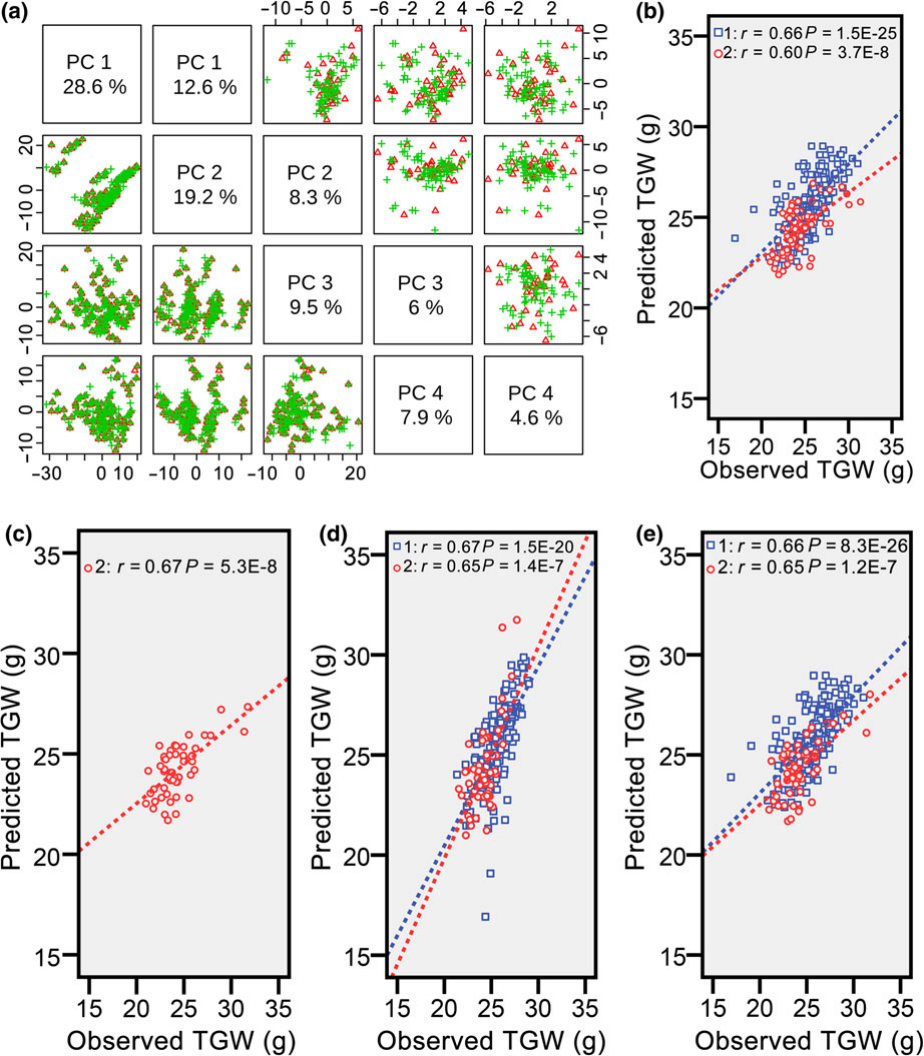
Prediction of thousand grain weight with core hybrids as the training set. (a) Distribution of core hybrids and “non‐core” hybrids. PCA was performed on predictive analytes, which were calculated for hybrids from both populations. The selected core hybrids are indicated by red triangles, and “non‐core” hybrids are indicated by green crisscrosses. (b) Correlation between the observed and predicted thousand grain weight with core hybrids from both populations (3N_2012_ & 3N_2015_) as the training set. Predictabilities of thousand grain weight for hybrids from populations in 2012 and 2015 with All_2012_ & 2N_2015_ (c), 2N_2012_ & 2N_2015_ (d) and 3N_2012_ & 2N_2015_ (e). These three different population structures are shown to further investigate the effect of population structures on predictability.

Because including different hybrids in the training set can change predictability, the predictability will be high when the correct hybrids are in the training set. One feature of these correct hybrids should be representativeness of roughly the entire population. To select core hybrids from both populations, we used scores of Principal Component 1 of hybrids in 2012 and 2015 in the PCA results (Table S4). These values were reordered from high to low, and one of every three hybrids was chosen according to the decreasing scores. The final numbers of chosen hybrids were approximately one‐third of the entire populations, and we named these hybrids 3N sets. To check representativeness of the 3N sets, we marked core hybrids in distribution plots of PCA results and found similar distribution trends in both populations (Figure 3a). Then, the 3N sets in 2012 (3N_2012_) and 2015 (3N_2015_) were combined as the training set (3N_2012_ & 3N_2015_), and the other “non‐core” hybrids were combined as the validation set. Finally, predictability of TGW for hybrids in 2015 reached 0.60 (*P *=* *3.7E‐8; Figure 3b), only slightly lower than that of 2012 (*r* = 0.66, *P *=* *1.5E‐25).

### Effect of population structures on predictability

Subsequently, to analyse the effect of population structures between training and validation sets on predictability; we adjusted numbers of core hybrids selected from both populations. First, changed numbers of core hybrids from the population in 2012 and a constant number of core hybrids in 2015 were combined to investigate the effect of numbers of core hybrids selected in 2012 on predictability. All, half, and one‐third of the hybrids from the population in 2012 and a half of the hybrids in 2015 were treated as the training set (All_2012_ & 2N_2015_, 2N_2012_ & 2N_2015_, and 3N_2012_ & 2N_2015_), and predictability of TGW for hybrids in 2015 reached the highest level (*r* = 0.67, *P *=* *5.3E‐8) with the All_2012_ & 2N_2015_ population structure (Figure 3c). The predictability only slightly decreased after removing half the number of hybrids from the population in 2012 from the training set (*r* = 0.65, *P *=* *1.4E‐7; Figure 3d). When further reducing the number of hybrids in 2012 from the training set, the predictability remained unexpectedly stable (*r* = 0.65, *P *=* *1.2E‐7; Figure 3e). Hence, the optimal number of core hybrids selected from the population in 2012 should be one‐third considering predictability changes and hybridisation tasks.

Then, we continued to identify an appropriate size for core hybrids selected from the population in 2015. With the same method described above, 2N_2015_, 3N_2015_, and 4N_2015_ sets were combined with All_2012_, 2N_2012_, and 3N_2012_ sets to form the training sets. Finally, the best combination was the population structure between training and validation sets with 3N_2012_ & 2N_2015_ (Table S5).

### An appropriate number of metabolic markers contribute to high predictability

**Figure 4:**
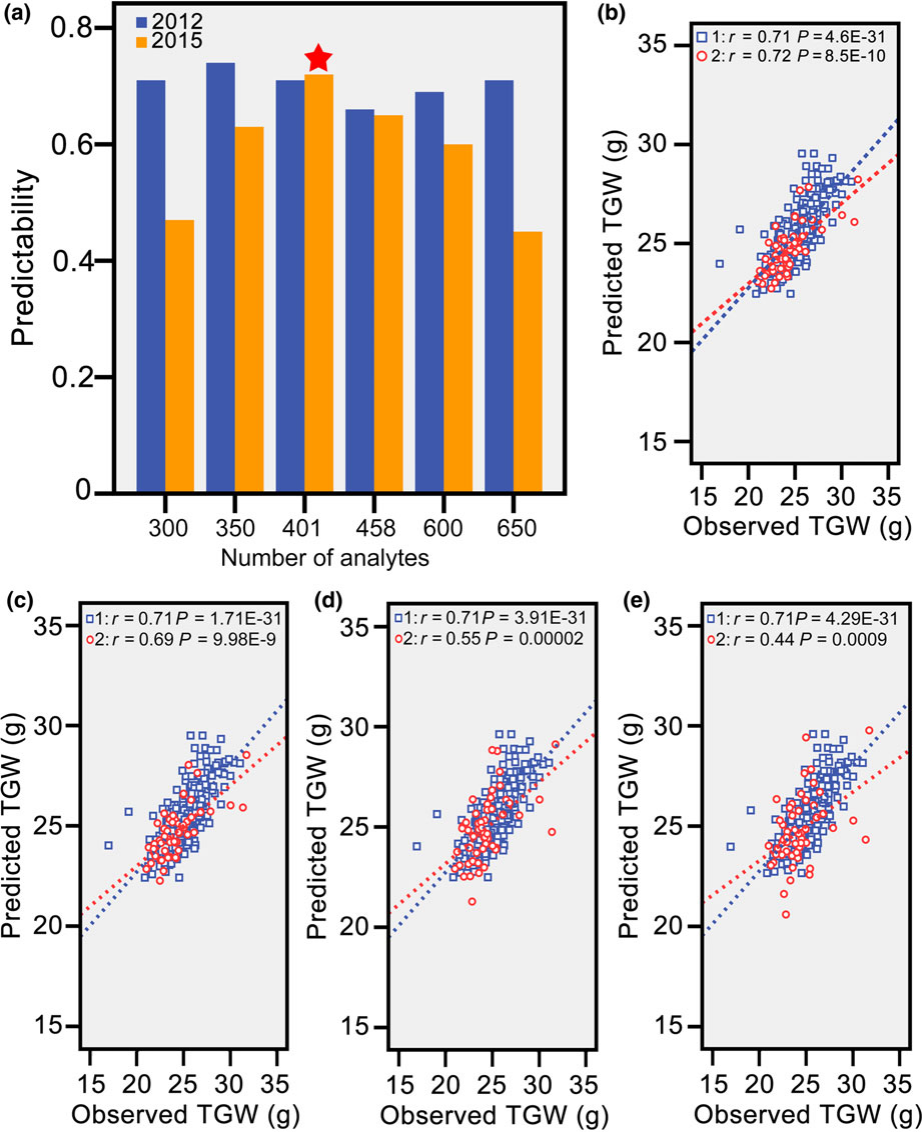
Effect of the numbers of metabolic markers on the predictability of thousand grain weight. (a) The relationship between the numbers of predictive analytes and predictabilities of TGW for hybrids from both populations. (b) Correlations between the observed and predicted thousand grain weights for hybrids from both populations with 401 metabolic markers. Based on the selected 401 analytes, PLS was conducted again to further filter low contribution analytes with VIP thresholds corresponding to 0.35 (c), 0.79 (d), and 1.0 (e).

To investigate the effect of numbers of predictive variables on predictability, different numbers of analytes (from 300 to 600), which were based on different thresholds of VIP values (from 1.3184 to 1.75), were selected to check variations of predictability with the 3N_2012_ & 2N_2015_ population structure. Unexpectedly, predictability of TGW for hybrids in 2015 peaked at 401 predictive variables (*r* = 0.72, *P *=* *8.5E‐10; Figure 4a) and was even higher than the predictability of TGW for hybrids in 2012 (*r* = 0.71, *P *=* *4.6E‐31; Figure 4b). Because 401 predictive analytes remained a large number, and Pearson correlation analysis indicated that many of these analytes were highly correlated (Figure S2), we performed the PLS regression analysis again to further filter low contribution analytes. After removing 100 analytes in the predictive model (VIP > 0.35), only a slight decrease was observed in the predictability for hybrids in 2015 (*r* = 0.69, *P *=* *9.98E‐9; Figure 4c). However, predictabilities were greatly reduced when the numbers of predictive analytes were reduced to 200 (VIP > 0.79, *r* = 0.55, *P *=* *0.00002; Figure 4d) and 158 (VIP > 1.0, *r* = 0.44, *P *=* *0.0009; Figure 4e). In summary, the prediction of TGW for hybrids across environments was achieved based on 300 metabolic markers with the 3N_2012_ & 2N_2015_ population structure.

## Discussion

Metabolites are good predictors of yield‐ or biomass‐related traits, disease resistance, and biotic/abiotic stress tolerance. Carbohydrate metrics can predict interyear biomass traits (*r* > 0.67) in *Miscanthus* (Maddison *et al*., 2017). Galactinol in maize leaf is a robust contributor to yield prediction under normal, drought, heat, and all other tested stress conditions (Obata *et al*., 2015). Moreover, proline and raffinose participate in drought and salt tolerance in both potato and rice (Sprenger *et al*., 2016; Wang *et al*., 2016; Zhao *et al*., 2014).

In this study, rice grain weight, which is a key component trait contributing to grain yield, was selected for prediction with metabolic markers (Figure S3a, b). To ensure robustness of predictive models across populations, cultivars including typical *indica*, intermediate types and typical *japonica* were selected to build the population in 2012 (Figure S3c). This design can decrease predictive errors and identify a predictive strategy for both intra‐ and inter‐subspecies hybrids. Cross‐validation is another crucial procedure in prediction, and overestimated predictabilities usually occur when hybrids in the training and validation sets have close relatedness. Thus, a new hybrid population in 2015 with distant relatedness was used to test the predictive model. Our results indicated that distant relatedness and environmental discrepancies severely affected the predictability of TGW. Then, hybrids from the new environment were included in the training set to neutralise these negative factors. Similar to previous findings, the predictability in the mixed predictive models benefited (Lado *et al*., 2013; Windhausen *et al*., 2012). In addition, scores of Principal Component 1 in PCA results were used to screen core hybrids for each population (Figure S3d, e). Finally, based on our results (Figure 4), this strategy was workable for prediction of TGW across environments in rice (Figure S3f).

The numbers of predictive variables (analytes) influence predictability (Sprenger *et al*., 2018; Xu *et al*., 2016, 2017). A total of 3746 analytes were detected in this study, but not all analytes were predictive of TGW. Values of VIP for Component 1 in the PLS‐DA result were used to filter TGW‐unrelated or low contribution analytes. Predictabilities of TGW for hybrids from the population in 2015 varied from low (*r* = 0.47, *P *=* *3.36E‐4) to high (*r* = 0.72, *P *=* *8.52E‐10) and then back to low (*r* = 0.45, *P *=* *8.14E‐4) with the increasing numbers of metabolic markers, which is similar to results with SNPs, transcripts or metabolites as predictive markers (Guo *et al*., 2016; Slavov *et al*., 2014). At last, after further removing low contribution analytes and additional PLS regression analysis, three hundred was the most appropriate number of analytes to predict grain weight for rice hybrids across environments, which indicated that a certain number of predictive analytes is necessary for high predictability. Furthermore, metabolite levels in hybrids can also show heterosis (overdominance); thus, the predictive variables that were mean relative abundances between corresponding parents might be limitations for the accuracy of predictions in this study.

Parental seedlings used for metabolomic analysis were grown under consistent experimental conditions and harvested on the 15th day in this study. Sampling at early developmental stages can reduce both sampling errors (compared to other later stage tissues, such as leaf or panicle) and experimental costs (Fernandez *et al*., 2016). Consistent with findings in other crops, metabolic profiles of young maize roots grown under controlled condition can predict hybrid performance across multiple fields (de Abreu *et al*., 2017). In addition, a few metabolic makers sampled at an early cultivation stage can predict tomato drought tolerance across diverse environments (Sprenger *et al*., 2018). Therefore, metabolite markers identified from early developmental stage tissues provide a new strategy for across‐stage and across‐generation predictions. Since other agronomic traits (grain number, tiller number or grain yield) have low heritability and are much more complicated than grain weight, strategies for accurate predictions of these complex traits require further explorations.

## Experimental procedures

### Plant materials

Two rice F_1_ hybrid populations were used in this study, and phenotypic thousand grain weight data were collected in two separate years. For the first population, eighteen representative *indica* and *japonica* rice inbred lines were chosen as the parents, and a complete diallel cross‐design was adopted in the hybridisation procedure as described previously (Dan *et al*., 2016). Seedlings were planted at the Hybrid Rice Experimental Base of Wuhan University in Ezhou, Hubei Province, China, in 2012. Three replicates were designed, and plants were planted with 16.5 × 26.4 cm spacing. Mature grains from five plants of each repeat were combined to measure thousand grain weight as previously reported (Dan *et al*., 2015). For the second population, 106 recombinant inbred lines (F_5_) from crosses between some of the 18 inbred lines and other inbred lines were selected as male parents, and a Honglian‐type cytoplasmic male sterile line Yuetai A was the female parent. Seedlings were planted in a new field at the Hybrid Rice Experimental Base of Wuhan University in Ezhou in 2015. Three replicates were designed, and plants were planted with 16.5 × 16.5 cm spacing. Thousand grain weights were collected according to the abovementioned method. Phenotypic data of thousand grain weight are given in Table S1.

Approximately 15 seeds of the 18 inbred lines, 106 recombinant inbred lines, and Yuetai A were sown in plastic pots with soil in a 2 × 2 cm spacing. Two replicates were designed for each line, and the pots were placed in a phytotron set at 30 °C and 70% humidity with an 8 h light/16 h dark photoperiod. Water was added to keep the soil moist. Since tissues close to soil may be contaminated, approximately 0.5 cm of tissues near the soil was removed with scissors. In addition, the remaining aboveground parts of approximately ten seedlings were washed with ddH_2_O three times before freezing in liquid nitrogen on the 15th day. For the 18 inbred lines, two repeats were treated as two biological samples. For the recombinant inbred lines and Yuetai A, two repeats were combined as one biological sample.

Seedling samples were ground into powder with liquid nitrogen, and 80 mg of homogenised powder was transferred into new 2‐mL EP tubes. One milliliter of precooled extraction liquid (methanol/acetonitrile/water, 2/2/1, v/v/v) was added to each tube and vortex‐mixed for 60 s. Tubes were placed at −20 °C for 60 min after two 30‐min ultrasonic treatments. The samples were centrifuged at 14 000 ***g*** for 15 min at 4 °C, and the supernatants were dried in a vacuum concentrator. Next, 100 μL of acetonitrile (acetonitrile/water, 1/1, v/v) was added to each sample. After vortex mixing, tubes were centrifuged at 14 000 ***g*** for 15 min at 4 °C, and the supernatants were used for liquid chromatography‐mass spectrometry analysis.

### LC‐MS/MS analysis

Ultra‐high performance liquid chromatography‐quadrupole time‐of‐flight mass spectrometry (UHPLC‐Q‐TOF MS) analysis was performed using a UHPLC system (1290 Infinity LC system, Agilent Technologies, Santa Clara, CA) coupled to a quadrupole time‐of‐flight mass spectrometer (Triple TOF 6600, AB SCIEX, Foster City, CA). A 2.1 mm × 100 mm Acquity UPLC HSS T3 1.8 μm column (Waters, Milford, MA) was used for RPLC separation. Solvent A was 0.5 mm ammonium fluoride (Sigma‐Aldrich, St. Louis, MO) in water, and acetonitrile (Merck, Whitehouse Station, NJ) was solvent B. The gradient of the mobile phase began with 1% B for 90 s, linearly increased to 99% in 11.5 min and kept constant for 3.5 min. Solvent B was reduced to 1% in 6 s, and a 3.4‐min re‐equilibration period was employed. Samples were placed in an automatic sampler at 4 °C and arranged in random order. The sample injection volume was 2 μL. The flow rate was 0.3 mL/min, and the column temperatures were held constant at 25 °C. Quality control samples were set per nine samples. The ESI source conditions were set as follow: ion source gas1 as 40, ion source gas2 as 80, curtain gas as 30, source temperature at 650 °C, and ion spray voltage floating at −5000 V in negative mode. The *m/z* range 60–1000 Da was set for MS only acquisition, and the accumulation time for TOF MS scan was set at 0.20 s/spectra. In auto MS/MS acquisition, the instrument was set to acquire over the *m/z* range 25–1000 Da, and the accumulation time for product ion scan was set at 0.05 s/spectra. The product ion scan was acquired using information dependent acquisition (IDA) with high sensitivity mode. IDA was set as follows: exclude isotopes within 4 Da, candidate ions to monitor per cycle: 10. The collision energy was fixed at 35 ± 15 eV, and declustering potential was set as −60 V.

Raw MS data (wiff.scan files) were converted to MzXML files using ProteoWizard MSConvert and processed with XCMS Online (Gowda *et al*., 2014a) for feature detection, retention time correction, and alignment. In the extracted ion features, only variables with more than 50% of nonzero measurement values in at least one group were retained. Metabolites were identified by accuracy mass (<25 ppm) and MS/MS data, which could match with local standards database.

### Processing of metabolomic data before analysis

Metabolomic data were prepared according to recommendations (Table S2; Fernie *et al*., 2011). The MetaboAnalyst (Xia and Wishart, 2016; Xia *et al*., 2015; www.metaboanalyst.ca) web‐based system was used for metabolomic data processing and analyzing. First, PCA was conducted to unstandardised metabolomic data to check instrument stability. Figure S4a shows that the quality control samples grouped together tightly, and the abundances of all detected analytes between the first and last quality control samples demonstrated highly significant correlations (Figure S4b). Most of all detected analytes (79.71%, *n* = 3746) of the quality control samples had a relative standard deviation of less than 0.2 (Figure S4c). Then, in data normalisation procedures, normalisation by the sum, none, and autoscaling were selected for sample normalisation, data transformation and data scaling respectively. Normalisation results are shown in Figure S5.

### Selection of metabolic markers for grain weight and core hybrids from populations in 2012 and 2015

Average relative abundances of detected analytes in parents were calculated as predictive analytes for each hybrid from the population in 2012, and corresponding large and small TGW hybrids were combined for a partial least squares‐discriminate analysis. Predictive ability of the model (*Q* ^2^) is demonstrated in Figure S6a, with *Q* ^2^ > 0.6. In addition, permutation testing showed that the *P*‐value was less than 5E‐04 (Figure S6b). Then, VIP values of the first component (Component 1) were used to determine putative metabolic markers for TGW (Table S3).

Principal component analysis was conducted on the calculated average relative abundances that were assigned to each hybrid from both populations. Scores of the first principal component (PC1) for each hybrid were used for choosing core hybrids (Table S4). The values were reordered from high to low and corresponding hybrids were selected every two, three or four intervals.

### Statistical analyses

Partial least squares regression analysis between mean parental relative abundances and thousand grain weight of hybrids was conducted in the IBM SPSS statistical software package, version 20.0 (IBM, New York, NY). The suitable number of latent factors was determined when the adjusted *R* ^2^ peaked. Then, the number of latent factors was fixed, and PLS was conducted again. The coefficient of each independent variable and the constants were used to build equations. Pearson correlation was performed between the observed and predicted TGW values, and the correlation coefficient was regarded as predictability. Normalisation of metabolomic data and PCA and PLS‐DA were performed with the MetaboAnalyst web‐based system (Xia and Wishart, 2016; Xia *et al*., 2015).

## Conflict of interest

The authors declare no conflict of interest.

## Author contributions

ZWD, YGZ and WCH designed experiments. ZWD and WCH prepared plant materials. ZWD, YPC, YHX, JRH, JSH and GXY collected phenotypic data. ZWD and YPC performed LC‐MS/MS analyses and analysed data. ZWD, YPC and WCH wrote the manuscript. All authors approved the final manuscript.

## Supporting information


**Figure S1** Population relatedness of hybrids from populations in 2012 and 2015.

**Figure S2** Heat map of correlations between the 401 predictive analytes.

**Figure S3** A technology roadmap of metabolome‐based prediction strategy for rice grain weight.

**Figure S4** Quality assessment of metabolomic data.

**Figure S5** Normalisation results of metabolomic data.

**Figure S6** Cross‐validation and permutation testing for the PLS‐DA model.

**Table S1** Thousand grain weight of parental inbred lines and hybrids in this study.

**Table S2** Metabolomic data of parental inbred lines.

**Table S3** VIP values of each analyte in the PLS‐DA.

**Table S4** Scores of the principal components in the PCA.

**Table S5** Effect of population structures on predictability.
