# Supplementary figures and images for: A metabolome‐based core hybridisation strategy for the prediction of rice grain weight across environments
Source: Plant Biotechnol J. 2018 Nov 12;17(5):906–13. doi: 10.1111/pbi.13024 (PMC6587747; doi:10.1111/pbi.13024)

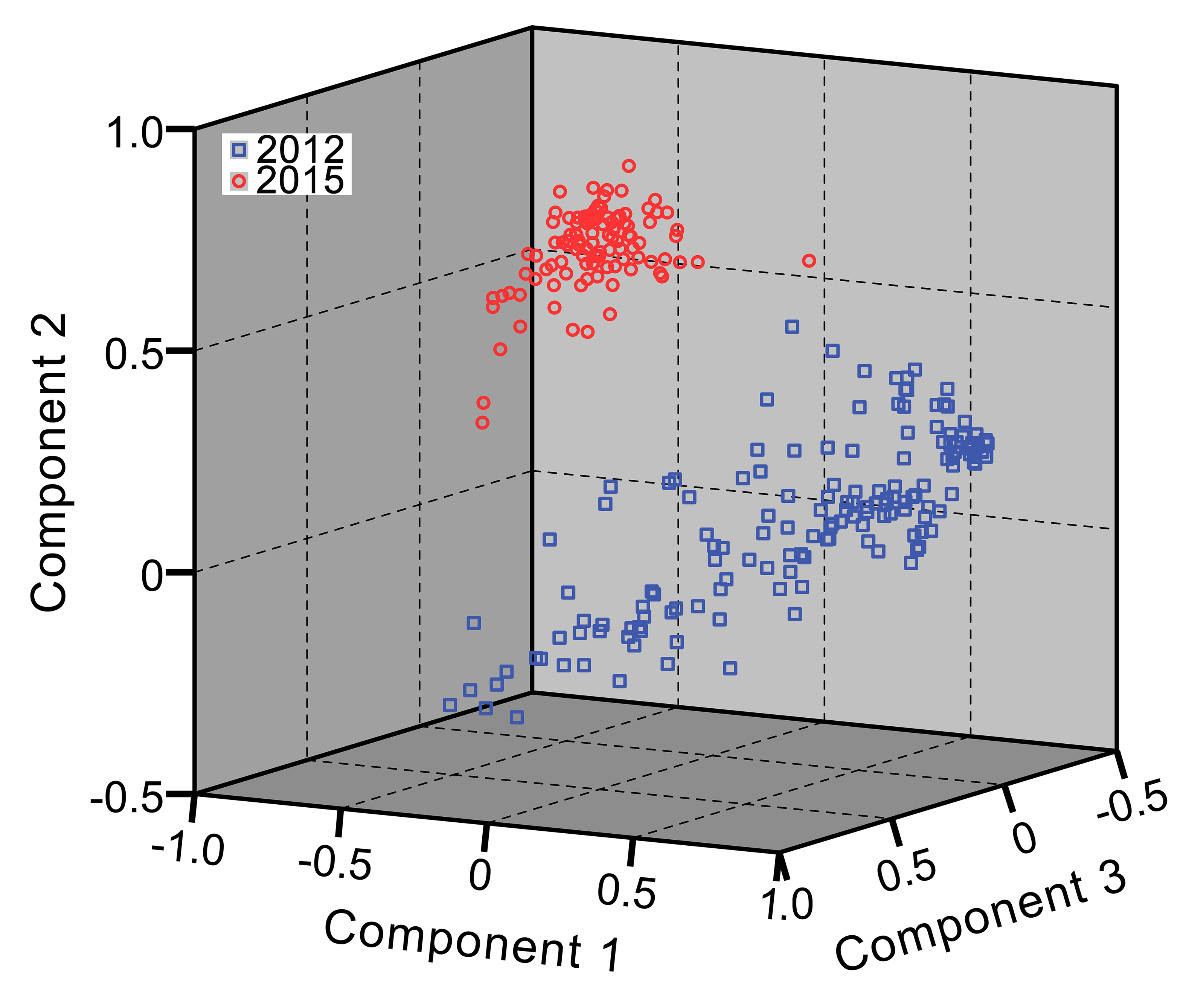

Supplement: Supplementary file 1 — Figure S1 Population relatedness of hybrids from populations in 2012 and 2015. [file PBI-17-906-s011.tif]

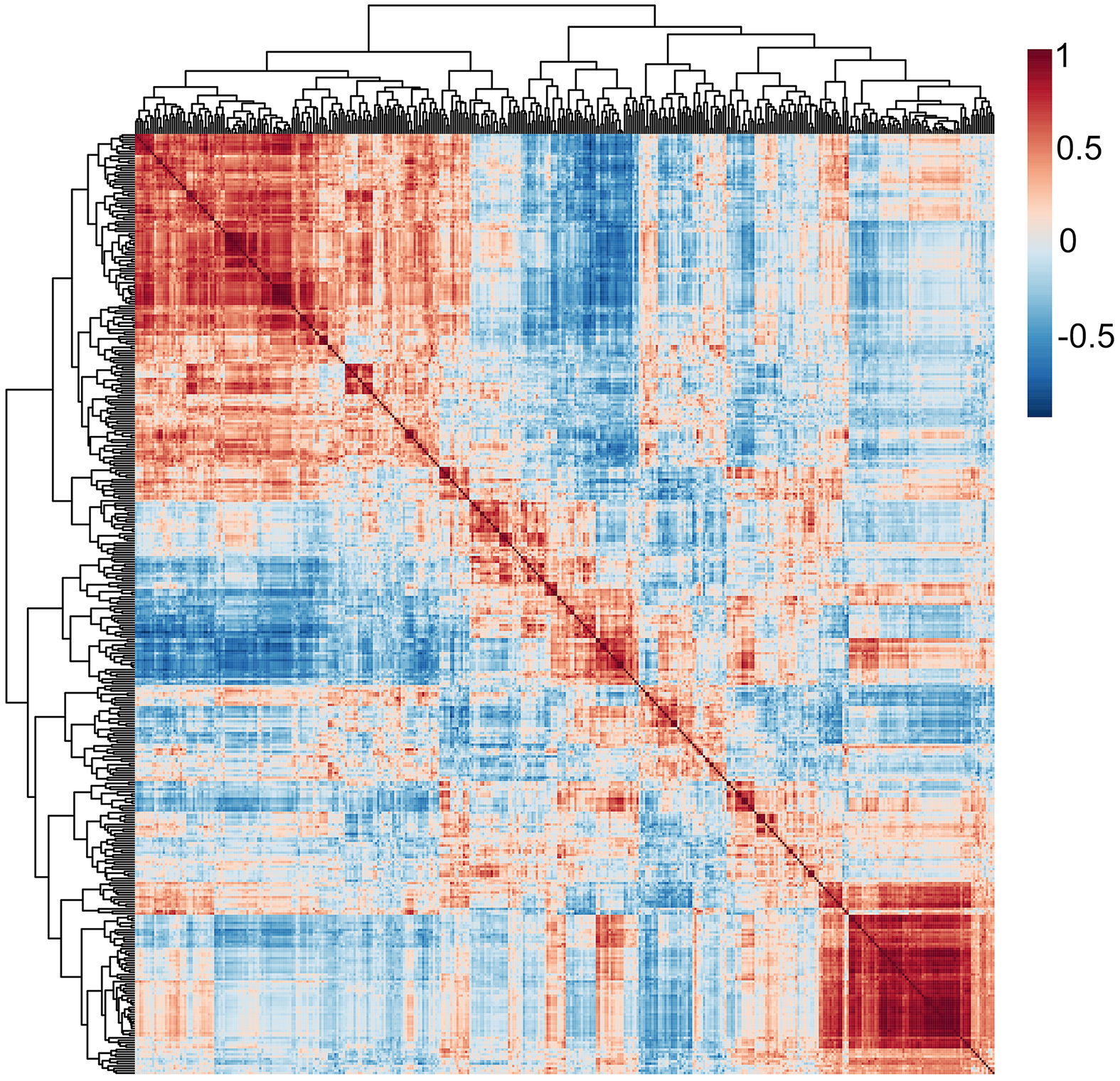

Supplement: Supplementary file 2 — Figure S2 Heat map of correlations between the 401 predictive analytes. [file PBI-17-906-s010.tif]

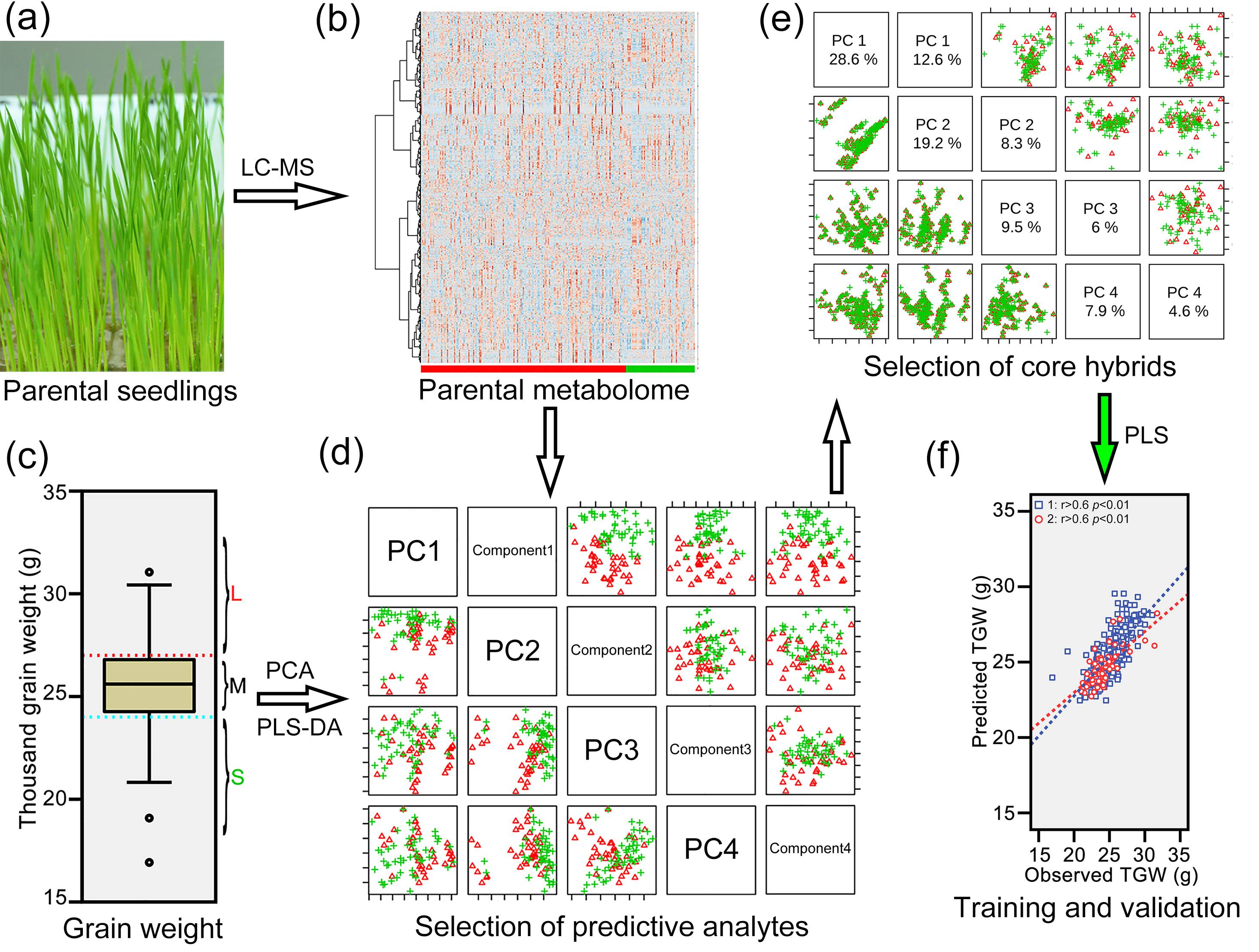

Supplement: Supplementary file 3 — Figure S3 A technology roadmap of metabolome‐based prediction strategy for rice grain weight. [file PBI-17-906-s009.tif]

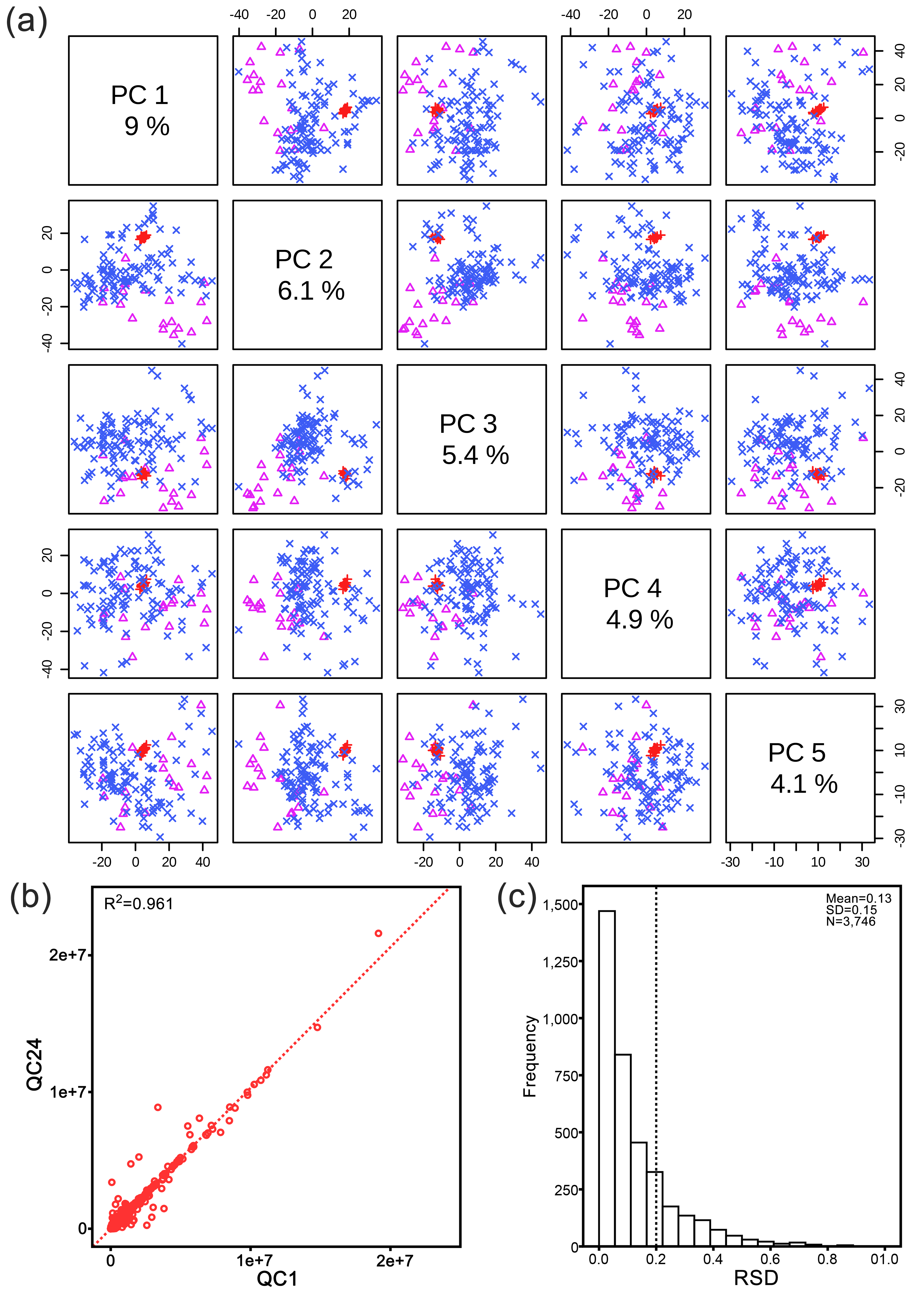

Supplement: Supplementary file 4 — Figure S4 Quality assessment of metabolomic data. [file PBI-17-906-s008.tif]

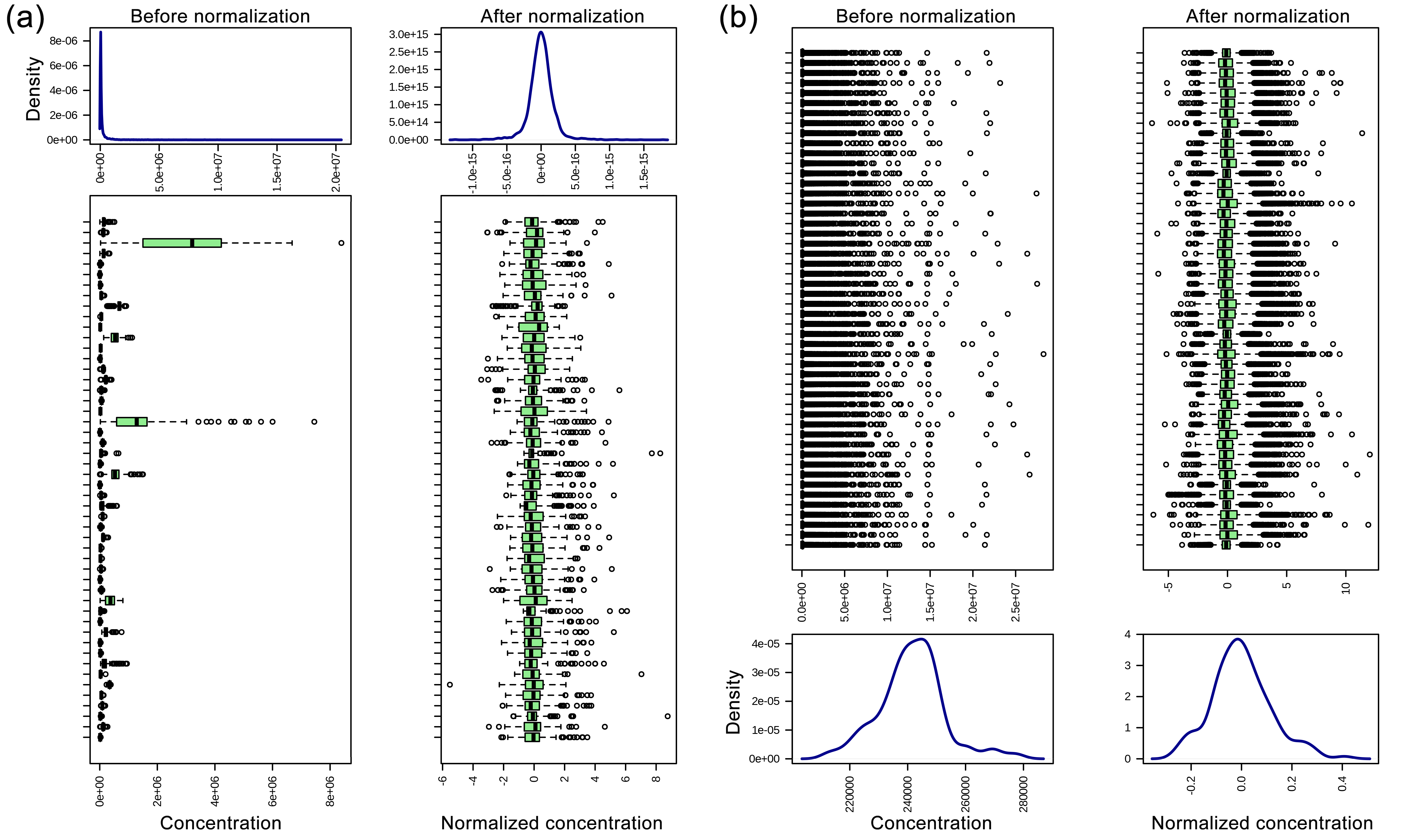

Supplement: Supplementary file 5 — Figure S5 Normalisation results of metabolomic data. [file PBI-17-906-s007.tif]

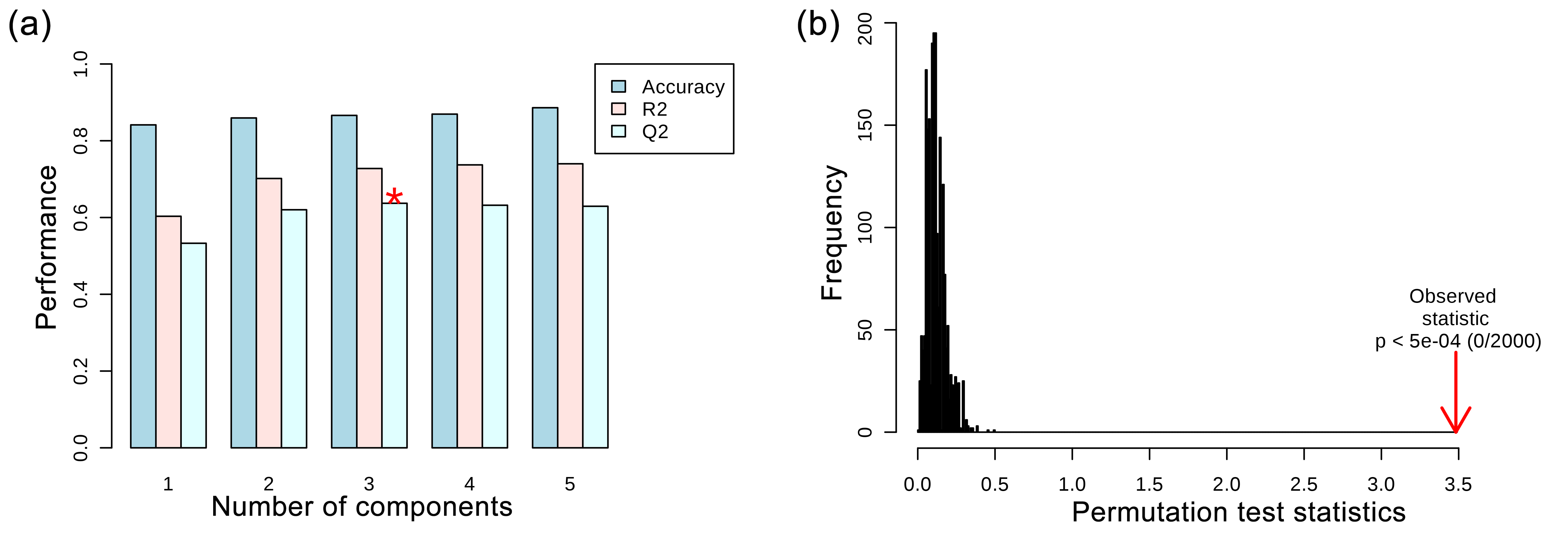

Supplement: Supplementary file 6 — Figure S6 Cross‐validation and permutation testing for the PLS‐DA model. [file PBI-17-906-s006.tif]
